# A Dual-Polarized Narrow-Beam Antenna for Microwave Interrogation of Back-Surface Flaws in Polyethylene Slabs

**DOI:** 10.3390/s26144361

**Published:** 2026-07-09

**Authors:** Ruonan Wang, Yong Li, Wenbin Ren, Pingjie Wang, Yang Fang, Zhenmao Chen

**Affiliations:** 1 State Key Laboratory for Strength and Vibration of Mechanical Structures, Shaanxi Engineering Research Centre of NDT and Structural Integrity Evaluation, School of Aerospace Engineering, Xi’an Jiaotong University, Xi’an 710049, China; w2502209459@stu.xjtu.edu.cn (R.W.); renwenbin@stu.xjtu.edu.cn (W.R.); wangpingjie@scsei.org.cn (P.W.); chenzm@mail.xjtu.edu.cn (Z.C.); 2 Sichuan Special Equipment Inspection Institute, Chengdu 610000, China

**Keywords:** microwave nondestructive testing, cross-polarized inspection, dual-polarized narrow-beam antenna, orthomode transducer, defect imaging

## Abstract

In view of the advantage of cross-polarized inspection (CrPI) in microwave nondestructive testing (MNT), in this paper a dual-polarized narrow-beam antenna as the pivotal reflectometric sensor is systematically designed and realized particularly for enhancement of detection and imaging of subsurface defects in dielectric structures. The antenna is equipped with a compact asymmetric waveguide orthomode transducer, with Teflon used as the internal filling material, in an effort to reduce its size and narrow the beamwidth. The internal dimensions of the realized dual-polarized narrow-beam antenna are optimized via numerical simulations. Based on the optimal design parameters, the antenna is fabricated and assessed through experiments. The experimental results reveal that the fabricated antenna has better metric indicators in terms of a return loss better than 10 dB, isolation better than 40 dB in 30.0 GHz~36.0 GHz and half-power beamwidths below 36.9° at 36.0 GHz. In order to further affirm the applicability of the fabricated antenna for CrPI, an MNT system is established to perform two-dimensional scanning and imaging of back-surface volumetric defects in polyethylene specimens. Based on the image characteristics of CrPI, a flaw-recovery algorithm is proposed to retrieve the defect opening profile. The averaged contrast-to-noise ratio of the processed CrPI-based image is found to be approximately five times larger than that of the raw CrPI-based image and fourteen times bigger than that of the raw CoPI-based image. Experimental results have further indicated that the fabricated antenna is feasible for not only co-polarized inspection (CoPI) but for CrPI, which exhibits higher testing sensitivity and defect-image contrast than CoPI. In conjunction with the flaw-recovery algorithm, by utilizing the dual-polarized narrow-beam antenna with the better metric indicators for CrPI, the image quality of the back-surface flaws in polyethylene slabs can be effectively improved.

## 1. Introduction

Microwave nondestructive testing (MNT) has been attracting considerable attention for nonintrusive interrogation and imaging of internal defects in dielectric structures due to its advantages of contactless inspection with a wide operating band, high testing sensitivity and ability to deeply penetrate dielectric materials [1,2,3,4,5]. It is noteworthy that the conventional reflectometric mode of MNT is co-polarized inspection (CoPI), where the incident and reflected waves have parallel electric-field polarization [6,7,8,9,10]. Although CoPI is found to be advantageous in terms of its relatively strong signal response and simple antenna structure, the signal response to the internal defect is usually masked by strong reflections from the structure surface, resulting in a sophisticated algorithm for separation of the response sources [11,12]. In an attempt to extract defect signals and simultaneously suppress background noise, cross-polarized inspection (CrPI) has been investigated. In CrPI, the incident and reflected waves have an orthogonal electric-field polarization. In the presence of a defect, the resulting structural discontinuity alters the polarization direction of the incident wave, bringing about the cross-polarized reflected wave component orthogonal to the incident electric field. In contrast, the flawless interface results in little variation in the cross-polarized signal [13,14]. The investigation of CrPI has been conducted particularly for the detection of cracks in the metal surface and fiber damage in composites [13,14,15,16]. A preliminary investigation of imaging subsurface volumetric defects in a glass fiber-reinforced polymer (GFRP) plate using a CrPI probe based on two rectangular waveguides has also been carried out [17]. However, studies regarding CrPI remain limited in terms of antenna performance and application for testing different types of defects. Previous investigations have been mostly focused on strongly depolarizing defects such as metal-surface cracks and fiber damage. However, the characterization and visualization of volumetric defects in dielectric components such as PE slabs still demands intensive scrutiny. Consequently, design and fabrication of the high-performance integrated CrPI antenna and analysis of characteristics of testing signals and defect images, along with the proposition of an image-quality enhancement algorithm, would push the boundary of CrPI for the detection, characterization and visualization of hidden flaws in dielectric structures, particularly in terms of testing sensitivity and defect-image quality.

An integrated antenna is deemed the pivotal component of an MNT system [18,19]. To the authors’ knowledge, most MNT probes are based on open-ended waveguides [10,11,12,13,14,15], while the horn antennas [20], dielectric-loaded antennas [21] and other near-field microwave imaging probes [22] have also been reported. Most of them are developed for CoPI. Even though dual-polarized antennas [13,14,15,16,23] have been employed for CrPI, the improvement of their performance in terms of broadband impedance matching, high port isolation and narrow-beam radiation characteristics still demands enhancement regarding testing performance. This motivates the development of an integrated dual-polarized narrow-beam antenna, especially for CrPI, to enhance the detection and visualization of defects in dielectric structures. The antenna design for CrPI is relatively tedious to ensure efficient radiation, high polarization purity and isolation. Abou-Khousa et al. proposed a dual-polarized microwave probe based on a circular waveguide and coaxial cable. Even at a frequency of 24.0 GHz, the probe exhibits good return loss and isolation [13,14,15]. Dvorsky et al. designed a septum-polarized-based circularly polarized waveguide antenna with a simulated isolation up to 20 dB in the 26.5 GHz to 40.0 GHz frequency band [16,23]. In an attempt to push the boundary of the CrPI antenna, the design of the advanced orthomode transducer (OMT) in wireless communication systems could be applied to MNT. This would benefit the design of a dual-polarized antenna that realizes not only CoPI but also CrPI, with improved metric indicators for frequency-swept testing.

As one of the critical dielectric materials, polyethylene (PE) has been widely used in engineering structures such as protective panels, corrosion-resistant liners, chemical storage tank linings and oil and gas transmission pipelines due to its corrosion resistance, good electrical insulation and broad engineering applicability [24,25,26]. During long-term service, hidden volumetric defects such as localized material loss may occur in PE components [27]. Such concealed defects pose a severe threat to structural integrity and safety. Therefore, in this paper, a dual-polarized narrow-beam antenna is designed and implemented specifically for microwave interrogation of back-surface volumetric defects in PE slabs via CoPI and, particularly, CrPI. Teflon is chosen to fill the internal section of the antenna, and it is extended beyond the aperture to minimize the size and enhance the directivity of the antenna. The parameters of the Teflon-filled part are optimized through simulations. Based on the design parameters, the dual-polarized narrow-beam antenna is subsequently fabricated. The metric indicators of the fabricated antenna are evaluated and compared with the typical probes presented in the literature. Using the fabricated antenna, an MNT system for testing and imaging back-surface volumetric flaws in a PE slab is built up. A flaw-recovery algorithm based on symmetry transformation and integral reconstruction is proposed for the production of the processed CrPI-based image. The feasibility and applicability of the antenna for CoPI and CrPI are further investigated.

## 2. Dual-Polarized Narrow-Beam Antenna

### 2.1. Structural Design and Analysis via Numerical Simulations

**Figure 1:**
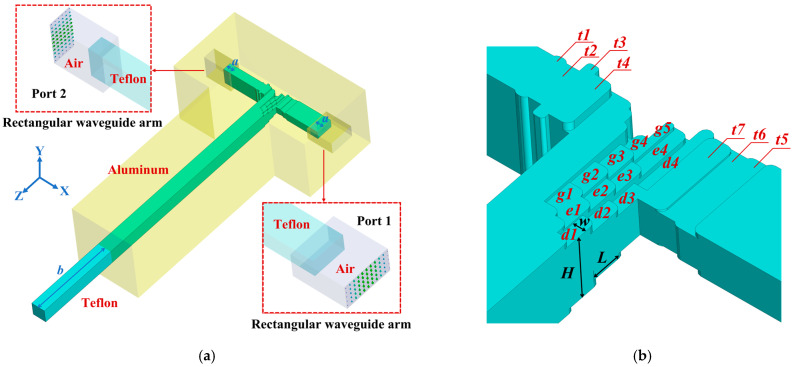
Simulation model of the proposed dual-polarized narrow-beam antenna. (**a**) Overall structure. (**b**) The OMT module.

In an effort to design and optimize the dual-polarized narrow-beam antenna, a simulation model is established in ANSYS HFSS 2024 R2 and illustrated in Figure 1. As can be observed in Figure 1, the antenna structure consists of two rectangular waveguide arms, a central T-shaped OMT module and a common square waveguide. The base material of the antenna body is aluminum, whilst the filling material is either Teflon or air. Note that in an effort to narrow the beam and facilitate antenna miniaturization, Teflon is filled into the internal section of the antenna and extended beyond the aperture with a certain length [18].

The OMT module is regarded as the key structure bridging the rectangular waveguide arms and the common square waveguide. In order to achieve the broadband impedance matching between the rectangular and square waveguides, the multi-section stepped impedance transformer [28] has been adopted in the OMT module of the antenna. The optimized dimensions of the OMT module after Teflon filling are summarized in Table 1, where *L*, *w* and *H* denote the length, width and height of each step in the OMT module, respectively. The OMT module effectively separates the two orthogonal modes from the reflected wave and transmits the isolated wave to the corresponding rectangular waveguide port. As a result, CoPI is readily implemented by using the same port for exciting and receiving the incident and reflected waves, respectively. In comparison, CrPI is realized with the excitation and reception of waves at the individual ports.

**Figure 2:**
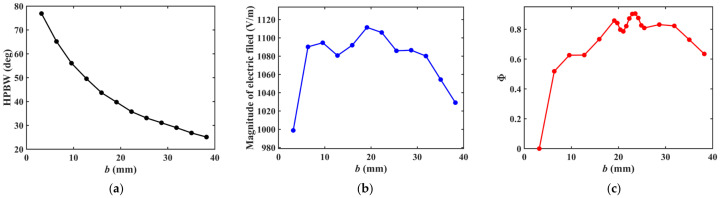
The correlation of *b* with: (**a**) the HPBW; (**b**) the magnitude of electric field; and (**c**) the metric indicator Φ.

The common square waveguide has a cross-sectional side length of 4.0 mm and is essentially supportive of wave propagation in the TE10 and TE01 modes over the Ka-band from 26.50 GHz to 36.58 GHz. In an attempt to narrow the radiation beam and enhance the wave directivity, the Teflon-filled section is extended beyond the square waveguide aperture with a length of *b*. Through a series of simulations, *b* is first optimized based on the trade-off among the directivity, radiated energy and sidelobe level. The optimization ensures that the antenna provides enhanced directivity, simultaneously retains sufficient radiated energy and keeps the sidelobe level within an acceptable range. The half-power beamwidth (HPBW) is used as a metric for directivity, while the radiated energy is characterized by using the electric-field magnitude. When Port 1 is employed for wave excitation from 29.0 GHz to 36.0 GHz, the variation of the E-plane HPBW against *b* is calculated and illustrated in Figure 2a. Note that *b* varies from 0.5*λ*_0_ to 6*λ*_0_ with an increment of 0.5*λ*_0_, where *λ*_0_ denotes the wavelength in the Teflon-filled section at the center frequency of 32.50 GHz.

It can be observed from Figure 2a that the HPBW decreases monotonically as *b* rises. This indicates that the main lobe of the radiation pattern can be narrowed by increasing *b*, thereby leading to the enhanced directivity of the antenna. Similarly, the correlation between the magnitude of the Y component of the electric field at the observation point 15 mm away from the center of the antenna aperture and *b* at the center frequency of 32.5 GHz is presented in Figure 2b. It is noticed from Figure 2b that the magnitude of the electric field increases as *b* increases, whilst it declines when *b* is approximately larger than 20 mm.

In an effort to seek the optimal *b*, a metric indicator Φ, which is a function of *b* and concerns the HPBW and electric-field magnitude, is proposed. It is written as:

(1) $$\left\{\begin{array}{l} \Phi \left(b\right)=\omega_{\Theta }\alpha \left(b\right)+\omega_{E}\beta \left(b\right),\;\omega_{\Theta }=\omega_{E}=0.5\\ \alpha \left(b\right)=\frac{\max\left[\Theta \left(b\right)\right]-\Theta \left(b\right)}{\max\left[\Theta \left(b\right)\right]-\min\left[\Theta \left(b\right)\right]},\;\beta \left(b\right)=\frac{E\left(b\right)-\min\left[E\left(b\right)\right]}{\max\left[E\left(b\right)\right]-\min\left[E\left(b\right)\right]}\; \end{array}\right.$$

where Θ(*b*) and *E*(*b*) denote the HPBW and electric-field magnitude varying with *b*, respectively. The electric-field magnitude is evaluated at the position 15 mm away from the center of the antenna aperture, which corresponds to the stand-off distance used in the experiments. *ω*_Θ_ and *ω_E_* are the weighting coefficients for the HPBW and electric-field magnitude, respectively. Note that: (1) a larger value of *α*(*b*) indicates a narrower HPBW and thus stronger directivity, while a larger value of *β*(*b*) corresponds to a higher electric-field magnitude at the observation point and thus a stronger radiated energy; and (2) the equal weighting coefficients (i.e., 0.5) are assigned to the HPBW and the electric-field magnitude because both quantities are equally vital for the antenna design. The narrower beamwidth is beneficial for improving lateral resolution and defect-image contrast, while a stronger electric-field magnitude is necessary to bring about a high amplitude of the reflected signal and signal-to-noise ratio. Therefore, the optimal value of *b* can be implicitly expressed as:

(2) $$b^{*}=\arg\max\left[\Phi \left(b\right)\right],\;\gamma <-10\;\mathrm{dB}$$

where b* stands for the optimized *b* sought via the trade-off among strong directivity, high radiated energy and reasonable sidelobe level. *γ* denotes the sidelobe level corresponding to the HPBW and is taken as the constraint. It should be pointed out that *b* varies with the selected sidelobe-level threshold. However, as long as the sidelobe level remains smaller than −10 dB [29], the optimized *b* is still determined by maximizing Φ. The computed Φ against *b* is exhibited in Figure 2c. It can be seen from Figure 2c that Φ hits the trough when *b* varies from 19.11 mm to 25.48 mm. Further analysis regarding the corresponding *b* reveals that the optimized length of the Teflon extended beyond the square waveguide aperture is 23.57 mm.

It is also noticeable from Figure 1 that each rectangular waveguide arm of the dual-polarized narrow-beam antenna for transmitting and receiving microwaves is essentially a rectangular waveguide, with the aperture width parallel to either the Y-axis or Z-axis. The modes of the microwaves through the rectangular waveguide arms involve microwaves with the polarization directions of the electric field along the Y- and Z-axes, respectively. In order to facilitate the connection of every rectangular waveguide arm with the standard waveguide-to-coaxial adapter for measurement, a short air-filled section is introduced at each rectangular waveguide port. Its cross-sectional dimension is 7.12 × 3.56 mm^2^. The remaining sections are filled with Teflon featuring the cross-sectional dimension of 4.90 × 2.45 mm^2^. In order to reduce the return loss, the Teflon-filled section of the rectangular waveguide is extended into the air-filled section with the length of *a*.

**Figure 3:**
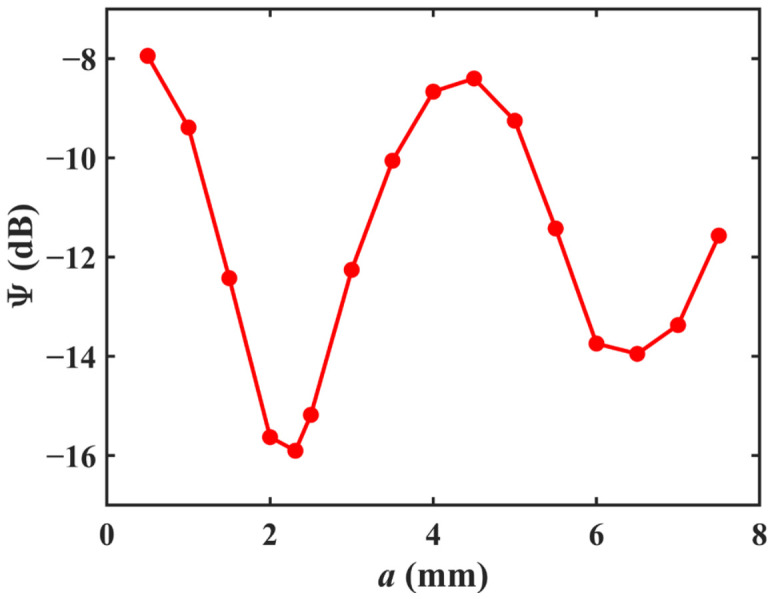
The correlation curve between Ψ and *a*.

In order to optimize *a*, the metric indicator Ψ as a function of *a* is thus formulated as:

(3) $$\Psi \left(a\right)=\frac{1}{2K}\sum_{n=1}^{K}\left[S_{11}\left(a,f_{n}\right)+S_{22}\left(a,f_{n}\right)\right]$$

where the scattering parameters, i.e., S_11_ and S_22_, characterize the return loss at Port 1 and Port 2, respectively. *f_n_* represents the *n*-th frequency sample over the operating frequency range of 29.0 GHz~36.0 GHz, *n* = 1, 2, …, *K*, where *K* = 701. The optimal *a* is written as:

(4) $$a^{*}=\arg\min\left[\Psi \left(a\right)\right]$$

where a* denotes the optimal *a*, which is obtained when the average response of two ports is minimized, indicating the optimal impedance matching between the air-filled and Teflon-filled sections of the antenna. It is noted that *a* changes from 0.5 mm to 7.5 mm with a step size of 0.5 mm. The resulting Ψ vs. *a* is portrayed in Figure 3. It can be observed from Figure 3 that Ψ reaches the minimum when 1.5 mm ≤ *a* ≤ 2.5 mm. The optimization solver in ANSYS HFSS is adopted to find the optimal value of *a* that minimizes Ψ, which ultimately gives *a* = 2.31 mm.

**Figure 4:**
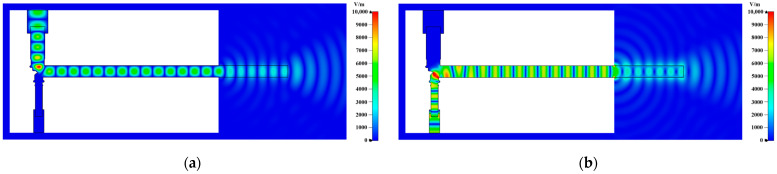
The simulated electric-field distributions at 36.0 GHz. (**a**) Port 1. (**b**) Port 2.

After all antenna parameters are determined, the simulated electric field distribution at the excitation frequency of 36.0 GHz is calculated and presented in Figure 4.

**Figure 5:**
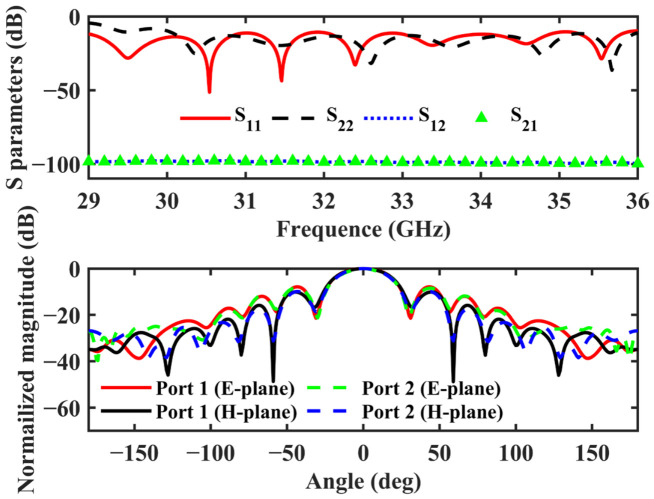
Simulated S parameters and radiation patterns at 36.0 GHz.

As shown in Figure 4, the microwaves with orthogonally polarized electric fields individually excited at two ports are transmitted into the square waveguide via the OMT module and radiated into the open space. The wave coupling between the two ports can barely be identified. Further simulations are focused on the return loss (characterized by S_11_ and S_22_), isolation (characterized by S_12_ and S_21_) and radiation patterns of the antenna. The results are portrayed in Figure 5.

It can be observed from Figure 5 that over the band from 30.0 GHz to 36.0 GHz, the return loss at each port is better than 10 dB, while the isolation is more than 95 dB. The HPBWs of Port 1 are 33.6° and 33.4° in the E-plane and H-plane, respectively, while those for Port 2 are 31.4° and 31.5°, respectively. The gains of Port 1 and Port 2 are 13.6 dB and 14.0 dB, respectively.

### 2.2. Performance Assessment of the Fabricated Antenna

**Figure 6:**
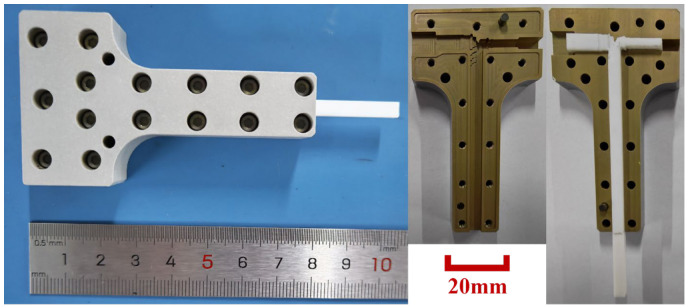
The overall picture and internal structure of the fabricated dual-polarized narrow-beam antenna.

**Figure 7:**
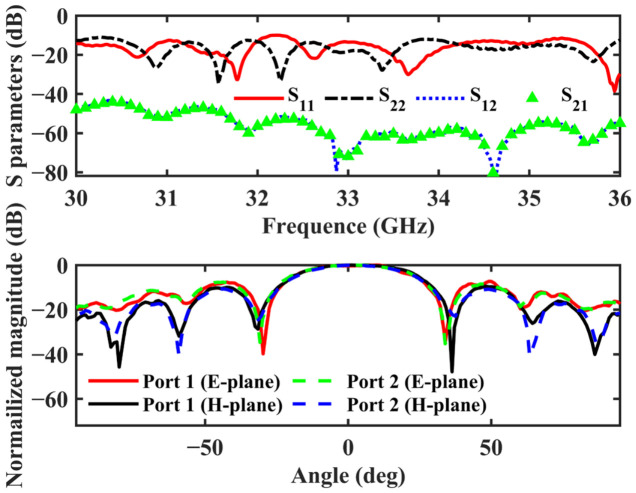
Measured S parameters and radiation patterns at 36.0 GHz.

Based on the optimized design parameters, the dual-polarized narrow-beam antenna is fabricated, and it is exhibited in Figure 6. The return loss and isolation are measured with the Keysight N5224A vector network analyzer (VNA). The radiation patterns are captured using the Keysight E8364B-50G VNA. The measurement results are shown in Figure 7. It is noticeable from Figure 7 that the measured return loss at both ports is more than 10 dB, whilst the isolation is higher than 40 dB over 30.0 GHz~36.0 GHz. Port 1 has HPBWs of 36.2° and 33.3° for the E-plane and H-plane, respectively, with a gain of 13.0 dB. In comparison, the HPBWs of 33.7° and 36.9° for the E-plane and H-plane, with a gain of 12.6 dB, can be identified for Port 2.

The performance of the antenna, especially for CrPI, with each probe reported in the literature is compared based on the metric indicators, including operating bandwidth, return loss and isolation. The comparison results are listed in Table 2.

It can be observed from Table 2 that even though the return loss of the fabricated antenna is slightly less than 20 dB, it remains better than 10 dB over the bandwidth of 6.0 GHz, together with the isolation twice larger than that reported in [13,14,15] or [16,23]. Besides the improved polarization isolation, the antenna also exhibits a more compact aperture size, with a side length of 4.0 mm. It should be pointed out that a narrow beam from the CrPI probe is favorable for enhancement of spatial resolution and mitigation of background interference. On account of the directivity-enhancement structure, the realized antenna achieves a measured gain of 12.60 dB and an HPBW of 36.9° at 36.0 GHz, while the reported probe in [13,14,15] exhibits only a simulated gain of 7.13 dB and an HPBW of 82.0° at 25.2 GHz. Moreover, the gain and HPBW of the probes in [16,23] were rarely presented. Therefore, the realized antenna could be advantageous for MNT with better metric indicators, including high isolation, compact size, enhanced directivity and narrow-beam radiation performance.

## 3. Experimental System and Imaging Results

**Figure 8:**
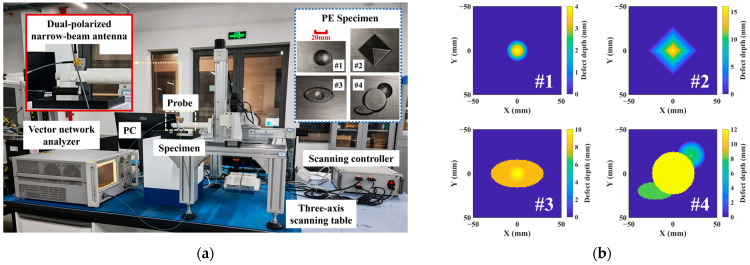
The MNT system for the inspection and visualization of the back-surface volumetric defect in the PE specimen. (**a**) The experimental system. (**b**) An illustration of the shape and size of volumetric defects in the PE specimen.

The fabricated dual-polarized narrow-beam antenna is employed for the inspection and imaging of the PE specimen subject to back-surface volumetric defects. The established MNT system is portrayed in Figure 8.

**Figure 9:**
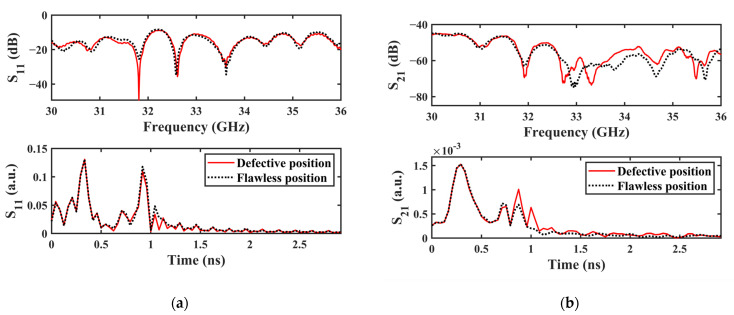
Experimental testing signals in frequency and time domains via: (**a**) CoPI; and (**b**) CrPI.

As can be observed in Figure 8, the system comprises the VNA (Keysight N5224A), a fabricated dual-polarized narrow-beam antenna, the PE specimen, a three-axis scanning table, a scanning controller and a computer. The thickness of the PE specimen is 20 mm. Defect #1 and Defect #2 are in the spherical-cap and pyramidal shapes and represent typical damage with smooth and sharp edges, respectively. Defect #3 comprises an elliptical flat-bottomed hole and a spherical-cap defect at the center, representing the composite anomaly. Defect #4 imitates the irregularly shaped damage. The fabricated antenna is mounted on the three-axis scanning table for 2D planar scanning of the specimen, with the scanning interval set to 2.0 mm. The VNA is swept from 30.0 GHz to 36.0 GHz, with a frequency step of 10 MHz. The reflected signals of S_11_ and S_21_ are measured at every scanning position for CoPI and CrPI, respectively, with the stand-off distance (between the bottom of the Teflon-filled section and specimen surface) of 15 mm. The spectral and temporal signals of CoPI and CrPI at the defective and flawless positions are exhibited in Figure 9.

It can be observed from Figure 9 that for CoPI, the reflected wave energy from the flawless position is slightly stronger than that from the defective position. In comparison, the reflected wave energy from the defective position is higher than that from the defect-free position with regard to CrPI. The testing sensitivity of each reflectometric mode is evaluated using the ratio of the difference between the total squared amplitudes of the temporal signals at the defective and flawless positions to the total squared amplitude at the flawless position. The calculated testing sensitivity for CrPI is 0.49, which is appreciably larger than that for CoPI (0.18). This indicates that: (1) the fabricated antenna is applicable for not only CoPI but also CrPI, and (2) CrPI is advantageous over CoPI, with considerably higher testing sensitivity to back-surface volumetric defects in PE structures. The enhanced testing sensitivity mostly resulted from the inherent capability of CrPI to suppress the background response, which is beneficial for improving the defect-to-background contrast in the resulting images.

**Figure 10:**
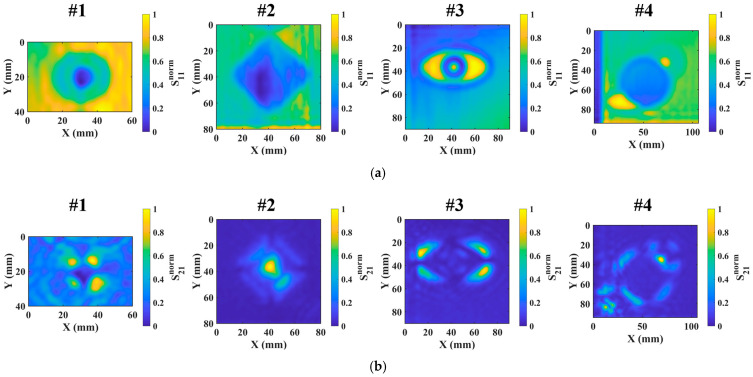
The acquired defect images via: (**a**) CoPI; and (**b**) CrPI.

Following 2D scanning, the synthetic aperture radar (SAR) imaging algorithm is subsequently applied to process the scanning data for defect-image production. One of the SAR imaging algorithms, the spectral reconstruction algorithm [30], is adopted to project the reflected signals at the stand-off plane (*z* = −*l*, where *l* denotes the stand-off distance) to the focal plane, i.e., the back surface of the PE specimen (*z* = *d*, where *d* is the specimen thickness). Note that the plane at *z* = 0 is the surface of the PE specimen. After the measured spectral signals of either CoPI or CrPI are transformed into the wavenumber domain using the two-dimensional spatial Fourier transform, the phase compensation of the resultant wavenumber spectrum is conducted by using the equation expressed as:

(5) $$F_{2}\left(k_{x},k_{y};z=d\right)=F_{1}\left(k_{x},k_{y};z=-l\right)⊙C_{1}\left(k_{x},k_{y}\right)⊙C_{2}\left(k_{x},k_{y}\right)$$

where the symbol ⊙ denotes the Hadamard product, namely element-wise multiplication. *F*_1_ and *F*_2_ denote the wavenumber spectrum at the stand-off plane and the focal plane, respectively. *k_x_* and *k_y_* are the wavenumber components along the X and Y directions. *C*_1_ and *C*_2_ denote the transfer functions corresponding to the air layer for the stand-off distance and the PE layer, respectively. They are formulated as:

(6) $$C_{1}\left(k_{x},k_{y}\right)=circ\left(\frac{k_{x}^{2}+k_{y}^{2}}{k_{1}^{2}}\right)\exp\left(j\sqrt{k_{1}^{2}-k_{x}^{2}-k_{y}^{2}}l\right);\;circ\left(\frac{k_{x}^{2}+k_{y}^{2}}{k_{1}^{2}}\right)=\left\{\begin{array}{l} 1,\;k_{x}^{2}+k_{y}^{2}<k_{1}^{2}\\ 0.5,\;k_{x}^{2}+k_{y}^{2}=k_{1}^{2}\\ 0,\;k_{x}^{2}+k_{y}^{2}>k_{1}^{2} \end{array}\right.$$

(7) $$C_{2}\left(k_{x},k_{y}\right)=circ\left(\frac{k_{x}^{2}+k_{y}^{2}}{k_{2}^{2}}\right)\exp\left(j\sqrt{k_{2}^{2}-k_{x}^{2}-k_{y}^{2}}d\right);\;circ\left(\frac{k_{x}^{2}+k_{y}^{2}}{k_{2}^{2}}\right)=\left\{\begin{array}{l} 1,\;k_{x}^{2}+k_{y}^{2}<k_{2}^{2}\\ 0.5,\;k_{x}^{2}+k_{y}^{2}=k_{2}^{2}\\ 0,\;k_{x}^{2}+k_{y}^{2}>k_{2}^{2} \end{array}\right.$$

where *k*_1_ stands for the equivalent wavenumber in the region between the antenna aperture and the specimen surface, whilst *k*_2_ denotes the equivalent wavenumber for the specimen. *k*_1_ = 4π*f*/*c* and $k_{2}=4\pi f\sqrt{\varepsilon_{r}}/c$, where *c* is the speed of light in free space. *f* denotes the excitation frequency. *ε_r_* is the relative dielectric constant of PE, and is set as 2.32, referring to [31] in the processing. The distribution of the reconstructed spectral signal over the focal plane is subsequently obtained by applying a two-dimensional inverse Fourier transform of *F*_2_. It is noted that: (1) in order to enhance the robustness of the defect image, the mean of the amplitude of the spectral signal reconstructed by SAR across all excitation frequencies is extracted as the signal feature for defect imaging; and (2) in an attempt to facilitate the comparison, every image is normalized by scaling pixel values to [0, 1]. The resultant defect images are portrayed in Figure 10.

It can intuitively be seen from Figure 10 that each defect on the back surface of the PE specimen can be detected in both the CoPI- and CrPI-based images. This implies that the dual-polarized narrow-beam antenna is applicable for not only CoPI but CrPI. Interestingly, in spite of the difference in defect visualization characterization between CoPI and CrPI [17], the CrPI-based image has less background noise and thus higher image contrast for defect detection than the image for CoPI. The stronger background noise in the CoPI-based image is mainly attributed to the characteristics of the reflected signal of CoPI. The signal is constituted by not only the scattering response induced by the defect but also the wave reflection from the specimen surface, which is highly sensitive to variations in stand-off distance [7]. Therefore, the CoPI-based image is appreciably more susceptible to background reflection and stand-off distance variation, incurring a higher background-noise level. In contrast, the reflected signal from CrPI originates from the depolarized scattering caused by defect-induced discontinuities, leading to a relatively weak background response and higher defect contrast [13].

Interestingly, by analyzing the CrPI-based image, it is noticeable that: (1) the recognized scattering points giving rise to the significant imaging response are predominantly distributed around the defect edges oblique to the polarization direction of the incident electric field; and (2) the localized defect-boundary section either parallel or perpendicular to the polarization direction of the incident electric field can barely be identified. This makes the defect visualization result via CrPI exhibit approximately symmetric imaging characteristics along these two directions. Such a phenomenon arises essentially from the response characteristics of the reflected signal of CrPI to the defect edge [14]. Specifically, the magnitude of the reflected signal of CrPI reaches the maximum when the localized defect-boundary section is deflected from the incident electric-field polarization direction at an angle of 45°. In contrast, it is nearly nulled for the scenario where the localized defect-boundary section has an angle of either 0° or 90° with respect to the incident electric-field polarization direction.

## 4. Flaw-Recovery Algorithm

**Figure 11:**
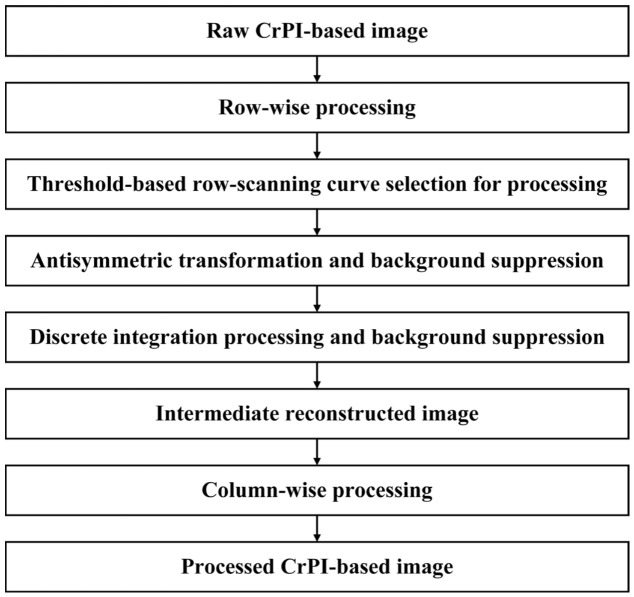
The flowchart of the proposed flaw-recovery algorithm.

It is inevitably noticed, particularly from Figure 10b, that due to the distinctive defect-visualized characterization of CrPI, the opening profile of each defect can barely be identified, even though the tangentially inclined defect edges are recognizable. In an attempt to mitigate the issue of defect visualization via CrPI, based on the symmetric characteristics of the detected flaw in the CrPI-based image, a flaw-recovery algorithm based on symmetry transformation and integral reconstruction is proposed to produce an image for identification of the defect opening profile. The flowchart of the proposed algorithm is shown in Figure 11.

Let the CrPI-based image be denoted as the matrix **I** with a size *r* × *u*, where *I*(*i*, *j*) represents the element in **I** at the index coordinates of row *i* and column *j*, respectively. The critical threshold *T* for separating the defect region from the background in **I** is determined using the OTSU algorithm [32]. For **I**, the key processing steps of the proposed algorithm are listed as follows.

(1) Derivation of the row-scanning curve via the row-by-row extraction of pixel values from the raw image. The row-scanning curve is defined as *g*(*j*), *j* = 1,2, …, *u*. When the maximum value of *g*(*j*) exceeds the threshold *T*, it is considered the curve corresponding to the defect response and thus is further processed. Otherwise, it is left unprocessed and deemed as the background.

(2) Derivation of the antisymmetric curve based on the row-scanning curve and background suppression. In consideration of imaging characteristics, the local minimum between two peaks sought in *g*(*j*) is regarded as the defect center, with the index coordinate of the column found to be *z_u_*. The starting and ending index coordinates of the column over the entire defect-related section in *g*(*j*) are identified with the threshold *T*. They are denoted as *p*_1_ and *p*_2_, respectively. The antisymmetric transformation is thus applied to *g*(*j*) to obtain the corresponding antisymmetric curve, which is expressed as:

(8) $$g^{*}\left(j\right)=\left\{\begin{array}{l} g\left(j\right),\;1\leq j\leq z_{u}\\ -g\left(j\right),\;z_{u}<j\leq u \end{array}\right.$$

where g*(*j*) stands for the antisymmetric curve. In the event that only a single peak is observed in the row-scanning curve, *z_u_* is set as the index coordinate of a column at the midpoint between *p*_1_ and *p*_2_. It is subsequently processed for background suppression, which can be formulated as:

(9) $$g^{\prime }\left(j\right)=\left\{\begin{array}{l} g^{*}\left(j\right)-\underset{1\leq j\leq p_{1}}{\min}\left[g^{*}\left(j\right)\right],\;1\leq j\leq z_{u}\\ g^{*}\left(j\right)-\underset{p_{2}\leq j\leq u}{\max}\left[g^{*}\left(j\right)\right],\;z_{u}<j\leq u \end{array}\right.$$

where *g*′(*j*) is the processed curve with the background suppression.

(3) Derivation of the discrete integral curve and background suppression. The discrete integration processing of *g*′(*j*) at each pixel location is conducted to obtain the resulting curve *G*(*j*), which can be written as:

(10) $$G\left(j\right)=\sum_{k=1}^{j}g^{\prime }\left(k\right)$$

The background suppression is subsequently performed for *G*(*j*). The resulting processed curve of *G*(*j*), i.e., *G*′(*j*), is acquired by the equation expressed as:

(11) $$G^{\prime }\left(j\right)=\left\{\begin{array}{l} G\left(j\right)-s_{1}\left(j\right),\;1\leq j\leq z_{u}\\ G\left(j\right)-s_{2}\left(j\right),\;z_{u}\leq j\leq u \end{array}\right.$$

where *s*_1_(*j*) and *s*_2_(*j*) are defined as:

(12) $$\;s_{1}\left(j\right)=\left\{\begin{array}{l} \underset{1\leq j\leq z_{u}}{\min}\left[G\left(j\right)\right],\;1\leq j\leq p_{1}\\ \frac{z_{u}-j}{z_{u}-p_{1}}\cdot \underset{1\leq j\leq z_{u}}{\min}\left[G\left(j\right)\right],\;p_{1}<j\leq z_{u} \end{array}\right.;\;s_{2}\left(j\right)=\left\{\begin{array}{l} \frac{j-z_{u}}{p_{2}-z_{u}}\cdot \underset{z_{u}\leq j\leq u}{\min}\left[G\left(j\right)\right],\;z_{u}\leq j\leq p_{2}\\ \underset{z_{u}\leq j\leq u}{\min}\left[G\left(j\right)\right],\;p_{2}<j\leq u \end{array}\right.$$

It is noted that the piecewise linear compensation in Equations (11) and (12) ensures the continuity of the processed curve. After the row-wise processing is completed with the aforementioned steps, an intermediate reconstructed image **P** with the matrix element of *G*′(*j*) is obtained.

(4) The column-wise processing, like the aforementioned procedures, is subsequently applied column by column for **P**, which ultimately brings about the final processed CrPI-based image. The key formulas used in the column-wise processing are written as:

(13) $$h^{*}\left(i\right)=\left\{\begin{array}{l} h\left(i\right),\;1\leq i\leq z_{r}\\ -h\left(i\right),\;z_{r}<i\leq r \end{array}\right.$$

(14) $$h^{\prime }\left(i\right)=\left\{\begin{array}{l} h^{*}\left(i\right)-\underset{1\leq i\leq p_{1}}{\min}\left[h^{*}\left(i\right)\right],\;1\leq i\leq z_{r}\\ h^{*}\left(i\right)-\underset{p_{2}\leq i\leq r}{\max}\left[h^{*}\left(i\right)\right],\;z_{r}<i\leq r \end{array}\right.$$

(15) $$H\left(i\right)=\sum_{k=1}^{i}h^{\prime }\left(k\right)$$

(16) $$H^{\prime }\left(i\right)=\left\{\begin{array}{l} H\left(i\right)-s_{1}\left(i\right),\;1\leq i\leq z_{r}\\ H\left(i\right)-s_{2}\left(i\right),\;z_{r}\leq i\leq r \end{array}\right.$$

where *s*_1_(*i*) and *s*_2_(*i*) are defined as:

(17) $$\;s_{1}\left(i\right)=\left\{\begin{array}{l} \underset{1\leq i\leq z_{r}}{\min}\left[H\left(i\right)\right],\;1\leq i\leq p_{1}\\ \frac{z_{r}-i}{z_{r}-p_{1}}\cdot \underset{1\leq i\leq z_{r}}{\min}\left[H\left(i\right)\right],\;p_{1}<i\leq z_{r} \end{array}\right.;\;s_{2}\left(i\right)=\left\{\begin{array}{l} \frac{i-z_{r}}{p_{2}-z_{r}}\cdot \underset{z_{r}\leq i\leq r}{\min}\left[H\left(i\right)\right],\;z_{r}\leq i\leq p_{2}\\ \underset{z_{r}\leq i\leq r}{\min}\left[H\left(i\right)\right],\;p_{2}<i\leq r \end{array}\right.$$

Note that in Equations (13)–(17), *h*(*i*), h*(*i*), *h*′(*i*), *H*(*i*) and *H*′(*i*) are the column-scanning curve, the antisymmetric curve of *h*(*i*), the background-suppressed curve based on h*(*i*), the discrete-integrated curve based on *h*′(*i*) and the background-suppressed curve based on *H*(*i*), respectively. *z_r_* represents the index coordinate of the defect center row in the column-scanning curve. The final processed CrPI-based image **Q** is essentially constituted by the matrix with the element of *H*′(*i*).

**Figure 12:**
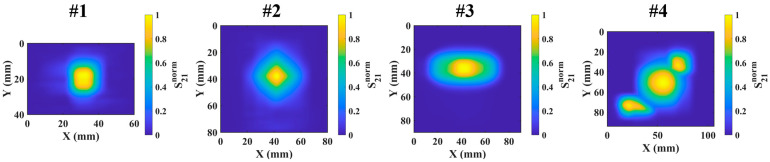
The processed CrPI-based image.

By applying the proposed algorithm, the processed CrPI-based image is presented in Figure 12. Note that $S_{21}^{\mathrm{norm}}$ in Figure 12 denotes the normalized *Q* (*i*, *j*).

**Figure 13:**
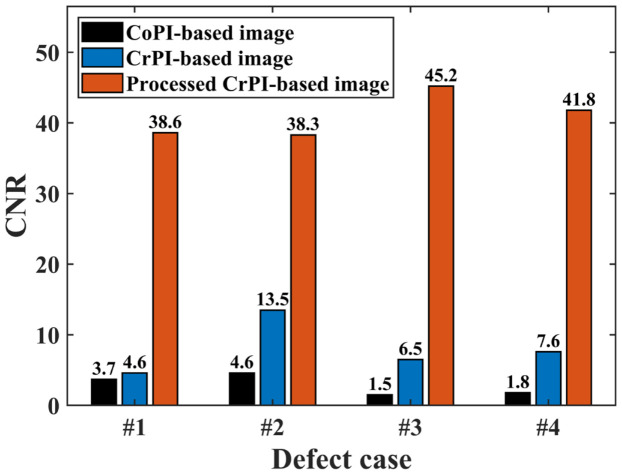
The CNR results for assessment of the imaging quality.

It can be observed in Figure 12 that the proposed algorithm is capable of producing an image in which the defect opening profile is intuitively recognized. The background noise is dramatically suppressed. In order to further evaluate the quality of the processed CrPI-based image obtained with the fabricated dual-polarized narrow-beam antenna alongside the proposed flaw-recovery algorithm, the contrast-to-noise ratio (CNR) [33] regarding each image in Figure 10 and Figure 12 is calculated with the equation expressed as:

(18) $$CNR=\frac{\left|\tau_{d}-\tau_{b}\right|}{\sigma_{b}}$$

where *τ_d_* and *τ_b_* denote the mean pixel value of the defective and background regions in a normalized image, respectively. *σ_b_* stands for the standard deviation of the pixel value in the background region. It is noteworthy that the defective and background regions are selected based on the known ground truth inferred in Figure 8b. The defective region is identified based on the known opening profile and parameters of the prefabricated defect, whilst the background region is the flawless area with relatively stable responses. The calculated CNRs for the raw CoPI- and CrPI-based images and the processed CrPI-based result are tabulated in Figure 13.

It is noticeable from Figure 13 that the CNR value corresponding to the processed CrPI-based image is considerably higher than that of the CoPI-based image. Further calculations indicate that the mean CNR of the raw CrPI-based image is more than twice that of the raw CoPI-based image, while the mean CNR of the processed CrPI-based image is roughly five times and fourteen times greater than those of the raw CrPI-based image and CoPI-based image, respectively. This further indicates that: (1) compared with CoPI, better defect-image contrast can be realized via CrPI; and (2) the fabricated dual-polarized narrow-beam antenna, together with the flaw-recovery algorithm, is capable of producing a defect image with improved image quality for volumetric defects in PE slabs.

## 5. Conclusions

A dual-polarized narrow-beam antenna especially for CrPI of MNT is designed and realized in this paper. The internal parameters of the antenna are optimized through a series of numerical simulations, achieving a return loss higher than 10 dB, an isolation greater than 95 dB in 30.0 GHz~36.0 GHz and HPBWs below 31.5° at 36.0 GHz. The fabricated prototype maintains a return loss better than 10 dB, an isolation level greater than 40 dB, and HPBWs below 36.9°. Based on the fabricated dual-polarized narrow-beam antenna, an MNT system is built up and used to perform two-dimensional scanning and imaging for back-surface volumetric defects in PE slabs. Experimental testing and imaging results indicate that the antenna is applicable for not only CoPI but CrPI, which exhibits higher testing sensitivity and defect-image contrast compared with CoPI.

Following the defect visualization, a flaw-recovery algorithm based on symmetry transformation and integral reconstruction is proposed based on the characteristics of the CrPI-based image. The defect opening profile can be readily identified by using the processed CrPI-based image, owing to the enhanced defect-image contrast. The mean CNR of the processed CrPI-based image is approximately five times and fourteen times larger than those of the raw CrPI-based and CoPI-based images, respectively. This further implies that a dual-polarized narrow-beam antenna is feasible, particularly for CrPI. The image quality of back-surface flaws in PE slabs can be enhanced by applying the realized antenna alongside the flaw-recovery algorithm.

Nevertheless, it should be pointed out that the testing results may be influenced by stand-off distance variation and by specimens with curved or rough surfaces and nonuniform thickness. This has opened up a door for further work including: (1) sophisticated algorithms for mitigating the influence of stand-off distance variation and anisotropic and heterogeneous characteristics of dielectric structures; (2) the implementation of more advanced flaw-recovery algorithms with high robustness for high-accuracy defect-image reconstruction; and (3) high-accuracy evaluation methods involving the localization and reconstruction of back-surface flaws in PE slabs.

## Figures and Tables

**Table 1:** Key dimensions of the dual-polarized narrow-beam antenna (unit: mm).

Steps	*L*	*w*	*H*	Steps	*L*	*w*	*H*
*d*1	1.59	0.69	3.45	*g*3	1.59	0.90	2.99
*d*2	1.59	0.69	2.66	*g*4	1.00	0.90	2.42
*d*3	1.59	0.69	2.09	*g*5	1.24	0.41	2.42
*d*4	4.42	0.69	1.52	*t*1	2.73	0.69	4.91
*e*1	1.59	0.69	3.66	*t*2	2.42	0.69	4.91
*e2*	1.59	0.69	3.22	*t*3	3.66	0.62	4.91
*e*3	1.59	0.69	2.64	*t*4	2.90	1.07	4.91
*e*4	2.76	0.69	2.07	*t*5	4.91	1.04	1.88
*g*1	1.59	0.90	3.66	*t*6	4.57	0.69	1.88
*g*2	1.59	0.90	3.56	*t*7	4.57	1.31	2.09

A chamfer of 0.17 mm is applied to *g*5 and *t*3, while a chamfer of 0.35 mm is utilized for the other steps.

**Table 2:** Comparison of the realized dual-polarized narrow-beam antenna with the reported probes.

Probe	[13,14,15]	[16,23]	Realized Antenna
Operating bandwidth	24.0 GHz	26.5 GHz~40.0 GHz	30.0 GHz~36.0 GHz
Return loss	14 dB	20 dB *	10 dB
Isolation	20 dB	20 dB *	40 dB
Antenna size	Inner diameter of 6.15 mm	Side length of 7.11 mm	Side length of 4.0 mm
Gain	7.13 dB at 25.2 GHz *	Not available	12.60 dB
HPBW	82.0° at 25.2 GHz *	Not available	36.9°

* The value is acquired from simulations in lieu of measurement.

## Data Availability

The data presented in this study are available on request from the corresponding author, subject to permission from an authorized person.
